# Effects of Parity and the Administration of Caffeine Plus PGF2α on Hormonal and Behavioral Changes During Parturition in Gilts and Sows

**DOI:** 10.3390/vetsci13080772

**Published:** 2026-08-01

**Authors:** Adrián Alejandro Corrales-Hernández, Patricia Roldán-Santiago, Héctor Orozco-Gregorio, Luis A. de la Cruz-Cruz, Ofelia Limón-Morales, Juan Manuel Vázquez García, Herlinda Bonilla-Jaime

**Affiliations:** 1 Departamento de Reproducción, Facultad de Medicina Veterinaria y Zootecnia, Universidad Nacional Autónoma de México, Avenida Universidad, Ciudad de México 04510, Mexico; adralexch@fmvz.unam.mx; 2 Ingeniería en Producción Animal, Universidad Politécnica de Francisco I. Madero, Tepatepec 42660, Mexico; horozco@upfim.edu.mx; 3 Departamento de Producción Agrícola y Animal, Universidad Autónoma Metropolitana, Calzada del Hueso 1100, Coapa, Villa Quietud, Coyoacán, Ciudad de México 04960, Mexico; ldelacruz@correo.xoc.uam.mx; 4 Departamento de Biología de la Reproducción, Universidad Autónoma Metropolitana, Unidad Iztapalapa, Ciudad de México 09340, Mexico; olm@xanum.uam.mx; 5 Universidad Autónoma Chapingo, Unidad Regional Universitaria de Zonas Áridas, Km 40 Carretera Gómez Palacio—Chihuahua, Bermejillo C.P. 35230, Mexico; dgips.invest34@chapingo.mx

**Keywords:** parity, caffeine, PGF2α, cortisol, estradiol, farrowing

## Abstract

Optimizing farrowing management is crucial in swine production, yet the combined effects of induction protocols and stimulants on the sow remain poorly understood. This study evaluated the effects of farrowing induction using cloprostenol as a single dose (full dose) or split dose, with or without caffeine administration, on the performance, behavior, and hormonal concentrations of primiparous (*n* = 25) and multiparous (*n* = 25) sows. Sows were assigned to five treatments: Control (*n* = 10), PGF2α Full Dose (175 μg) (*n* = 10), PGF2α Split Dose (87.5 + 87.5 μg) (*n* = 10), PGF2α Full Dose + Caffeine (420 mg) (*n* = 10), and PGF2α Split Dose + Caffeine (420 mg) (*n* = 10). The PGF2α Split Dose and PGF2α Split Dose + Caffeine treatments significantly reduced farrowing duration by up to 100 min in primiparous sows (122.40 ± 82.24 and 136.00 ± 38.33 min, respectively; *p* = 0.002), and in multiparous sows by up to 76 min (134.80 ± 73.84 and 158.00 ± 44.82 min, respectively; *p* < 0.05), compared to the Control and PGF2α Full Dose groups. Furthermore, the PGF2α Split Dose induction method lowered the frequency of stress-related behaviors (0.01 and 0.08 for PGF2α SD, and 0.03 and 0.08 for PGF2α SD + Caffeine, for gilts and sows respectively), with sows showing fewer posture changes and maintaining more stable resting positions, particularly the lying lateral posture (0.86 for PGF2α SD and 0.96 for PGF2α SD + Caffeine). Cortisol levels varied by parity, with multiparous sows showing higher prepartum and intrapartum levels, while primiparous sows peaked twelve hours postpartum; estradiol levels remained unaffected by treatments. In conclusion, inducing farrowing with a split-dose prostaglandin protocol is highly efficient, and the addition of caffeine further enhances farrowing performance while significantly improving behavioral welfare by reducing standing postures associated with discomfort in both primiparous and multiparous sows.

## Simple Summary

Research in swine production often focuses only on the piglets, leaving the health and performance of the sow largely overlooked. Additionally, while intravulvar caffeine is an authorized pharmacological compound in Europe, its specific effects on the sow during birth remain poorly understood. This study aimed to evaluate how the combined administration of caffeine and prostaglandin F2α affects the hormones, behavior, and farrowing performance of both primiparous and multiparous sows. The results show that combining caffeine with a split-dose hormone treatment is an effective strategy to improve farrowing performance and behavior. This combined treatment significantly shortened the total duration of farrowing and the birth intervals between piglet expulsions. It also reduced signs of distress, keeping the sows calmer and in more favorable resting positions. Finally, stress and reproductive hormone levels revealed that a sow’s physical response during birth depends on parity. Ultimately, these findings benefit society by improving animal welfare in agriculture and optimizing food production efficiency through safer, faster, and more comfortable births for farm animals.

## 1. Introduction

Caffeine has been shown to have beneficial effects on neonatal survival, vitality, and metabolic recovery [1,2], and its administration through the sow for subsequent distribution to the fetuses has been found to be highly effective [3]. However, in swine production research, emphasis has traditionally been placed on factors affecting the neonate, leaving aside the direct effects of the treatment on the sow itself; indeed, it has only been generally reported that there are no alterations in maternal behavior due to the effect of caffeine [4,5].

It is necessary to consider that other factors could modify the response to caffeine, including parity, which is known to influence the performance of both the sow and her offspring [6,7]. Likewise, under commercial swine farm conditions, farrowing induction using PGF2α or its synthetic analog, cloprostenol, is a common practice. Although this is performed to schedule staff availability for the efficient supervision of the sow and her offspring during farrowing, thereby indirectly increasing neonatal survival [8], the application of this exogenous hormone modifies the physiological course of gestation.

The pharmacological effect of caffeine is achieved through its stimulation of respiratory potency, neuronal contractility, and cyclic adenosine monophosphate (cAMP) concentrations [9]. However, to date, caffeine has only been evaluated as a standalone treatment, with research focused exclusively on the offspring; therefore, its potential effects on the farrowing performance and well-being of the sow remain unknown. Based on the above, the objective of the present study was to evaluate the effect of caffeine and PGF2α administration on the endocrine profiles, behavior, and farrowing performance of primiparous and multiparous sows.

## 2. Materials and Methods

This research was carried out in the northwestern zone of the State of Mexico, Mexico, within an intensive commercial pig farm. The climate typical of this area ranges from temperate to semi-cold, with sub-humid characteristics. The Institutional Subcommittee for the Care and Use of Experimental Animals (SICUAE) of the Universidad Nacional Autónoma de México reviewed and authorized all experimental protocols under the approval number CICUAE.DC-2021/3-1.

### 2.1. Housing and Environment

Sows were transferred to the farrowing facilities approximately seven days before their anticipated delivery date. Environmental conditions within the facility were characterized by a relative humidity spanning 46.4% to 73.4%, with minimum and maximum average temperatures recorded at 18.9 °C and 26.9 °C, respectively. The sows were individually housed in farrowing crates equipped with slatted plastic flooring, featuring an inner stall measuring 1.45 m long × 0.8 m wide × 0.57 m high, and provided with a plastic feeder and two metallic nipple drinkers. Sows were fed a conventional gestation diet adjusted to their nutritional requirements [10], at a rate of 2.5 to 3.0 kg/day, with ad libitum access to water. Upon entering lactation, the diet was switched to a corn- and soybean meal-based lactation formulation until day 21 postpartum.

Within the piglet creep area, a temperature averaging 38.5 °C was sustained using a 0.37 × 1.2 m heating mat enclosed by a plastic box.

### 2.2. Animals and Treatments

**Figure 1:**
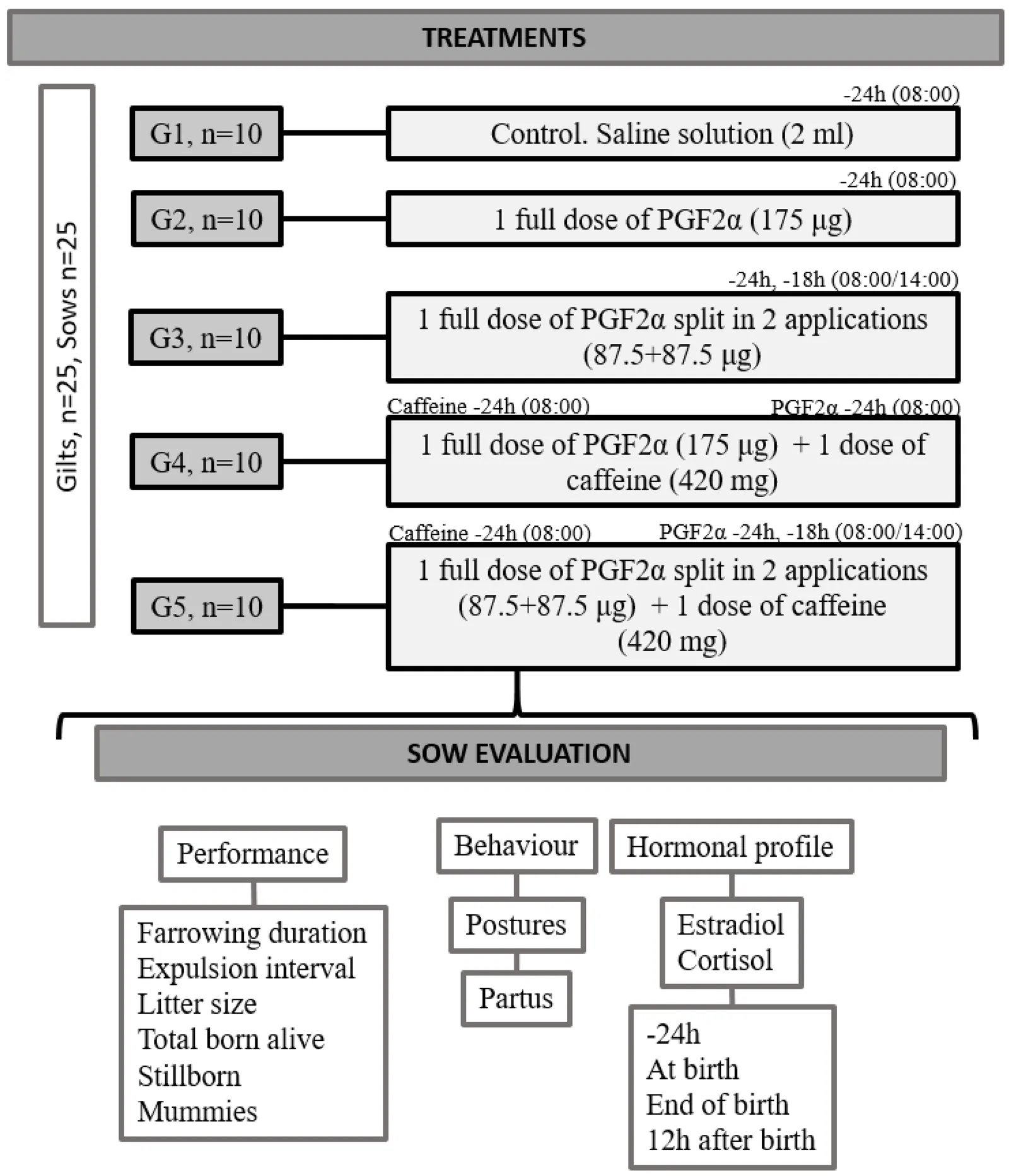
Description of the parameters evaluated and analyzed in gilts and sows. G: Group, Tx: Treatment, PGF2α: Prostaglandin F2α.

A total of 50 pregnant Yorkshire × Landrace sows were used: 25 primiparous and 25 multiparous (parity 2 to 4). Sows were randomly assigned to five study groups, with 5 primiparous and 5 multiparous sows per treatment. These groups were: G1 (Control), a negative control where females were administered 2 mL of physiological saline solution via intramuscular (IM) injection to simulate handling; G2 (PGF2α FD), sows that received a complete dose of prostaglandins for induction (175 μg cloprostenol); G3 (PGF2α SD), sows undergoing induction via a divided dose of prostaglandins (87.5 + 87.5 μg cloprostenol); G4 (PGF2α FD + Caffeine), sows induced with a full dose of prostaglandins plus caffeine administration (175 μg cloprostenol + 420 mg caffeine); and G5 (PGF2α SD + Caffeine), sows receiving a divided dose of prostaglandins complemented by caffeine administration (87.5 + 87.5 μg cloprostenol + 420 mg caffeine) (Figure 1). To synchronize farrowing, sows were treated with sodium cloprostenol (Celoprost^®^, ParFarm Laboratorios de Productos Veterinarios, México City, Mexico). For the Full Dose groups, a single 175 μg application was administered at 08:00 h, 24 h prior to the expected farrowing date. For the Split Dose groups, the total 175 μg dose was fractionated into two equal administrations of 87.5 μg. The initial application was performed at 08:00 h (24 h prior to the anticipated farrowing date), followed by the second application 6 h later at 14:00 h. This procedure was standardized by taking into account an expected average gestation of 115 days.

The farrowing induction protocol with PGF2α was adapted from the methodology described by Decaluwe et al. [8]. Anhydrous caffeine (Sigma-Aldrich, St. Louis, MO, USA; powder, ReagentPlus^®^) was used. For administration, the 420 mg dose was dissolved in an 8 mL vehicle composed of water and propylene glycol. This solution was administered to the sows in groups G4 and G5 via a deep submucosal injection into the vaginal vestibulum. The administered caffeine dose was selected in accordance with previous reports by Orozco-Gregorio et al. [1] and Sánchez-Salcedo et al. [11].

### 2.3. Sow Performance

The following reproductive variables were recorded for each sow: farrowing duration, defined as the time elapsed from the birth of the first to the last piglet [12]; total litter size; number of piglets born alive; and the number of stillborn and mummified fetuses per litter. Additionally, the birth interval was recorded as the time elapsed between the expulsion of consecutive piglets. Sows that required obstetrical intervention due to dystocia (e.g., manual assistance or the administration of exogenous uterotonics) were excluded from the study.

### 2.4. Sow Behavior

**Figure 2:**
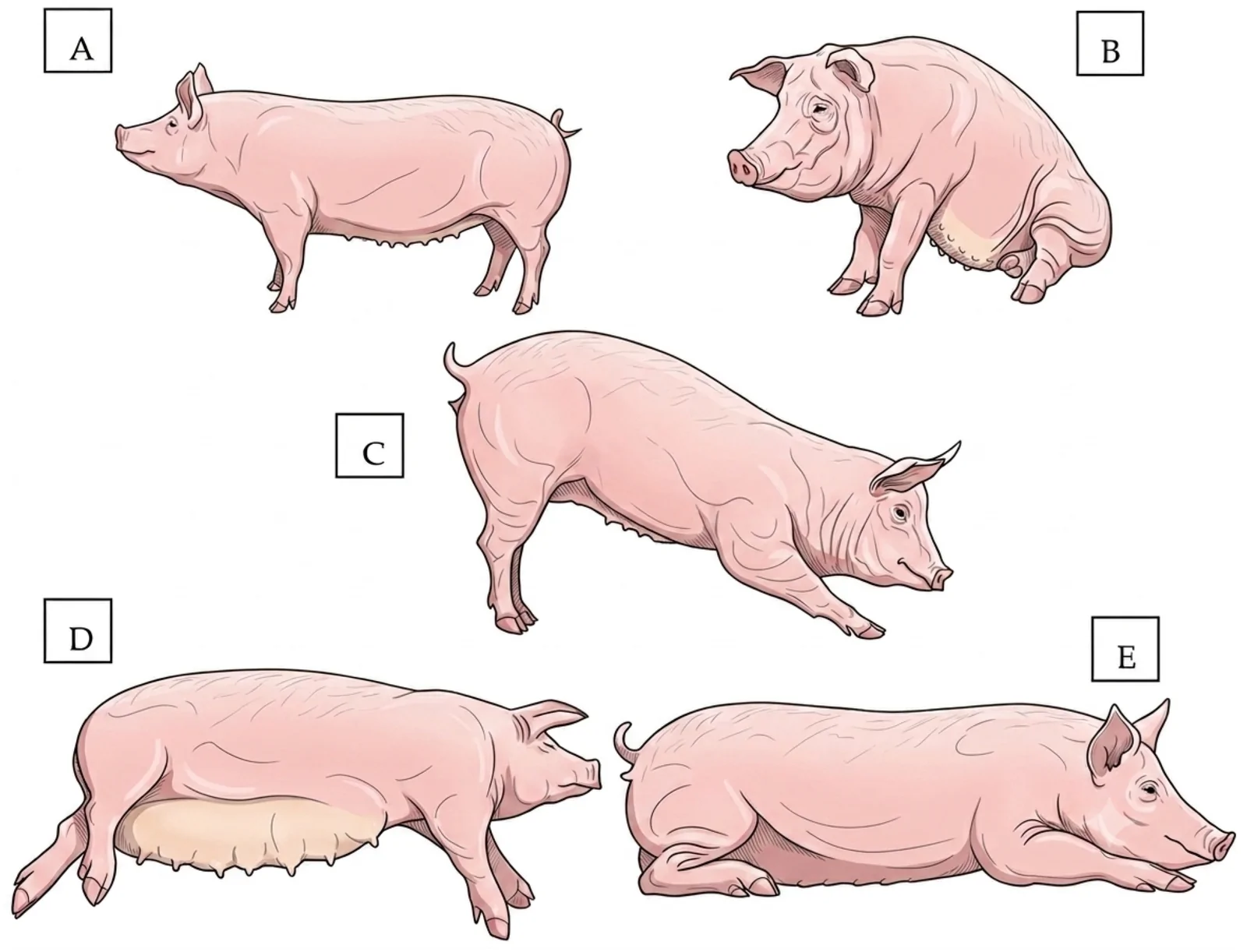
Graphical representation of the evaluated sow postures adapted from Ison et al. [13]. (**A**) Standing: upright posture with all four feet on the floor. (**B**) Sitting: partly erected on extended front legs with the hindquarters contacting the floor. (**C**) Kneeling: front knees on the floor with hind legs extended. (**D**) Lateral recumbency: lying down with one shoulder contacting the floor and the udder exposed. (**E**) Sternal recumbency: lying down with the chest and abdomen contacting the floor and front legs extended or folded under the body.

The farrowing process of each sow was recorded using closed-circuit television cameras (*DAHUA KITXVR1B04*, 2 Megapixels with audio and H.265+ compression) to subsequently evaluate the frequency of posture changes associated with distress in the sows. This evaluation began immediately upon the expulsion of the first piglet. Posture changes were assessed using instantaneous scan sampling at 5 min intervals during the first 150 min of farrowing, yielding a total of 30 discrete observations per sow. During each scan, the exact posture of the sow was recorded. The 150 min observation window was selected to standardize the assessment across all experimental groups, as it encompasses the average duration of the split-dose treatments. The ethogram and postures considered were based on those described by Ison et al. [13] (Figure 2).

### 2.5. Blood Sampling and Hormonal Evaluation

Blood sampling in the sows was performed via the mammary vein, following the methodology described by Scollo et al. [14]. This procedure involved venipuncture through the ventral abdominal wall, accessing the mammary vein in the space between the second and third mammary glands at a 90° angle. Blood samples were obtained at four specific time points: T1, 24 h prior to the expected farrowing date; T2, onset of farrowing; T3, completion of farrowing; and T4, 12 h post-farrowing. Because T2 and T3 corresponded to specific biological events, the exact time elapsed between T1 and the subsequent sampling points varied among individual sows. The onset of farrowing was defined as the expulsion of the first piglet, whereas farrowing completion was defined as the expulsion of the last piglet, excluding the placental expulsion period.

Restraint was not required during blood collection. Regardless of whether the sow was standing, sitting, or in lateral recumbency, all blood samples were drawn in less than 30 s to prevent alteration of the endocrine variables. Blood samples were collected using *BD Vacutainer*^®^ Serum Plus 10 mL tubes (Becton, Dickinson and Company, Franklin Lakes, NJ, USA) [14] and were centrifuged at 3000 rpm for 15 min. The extracted serum was stored at −70 °C until analysis. Serum cortisol and estradiol concentrations were determined via enzyme-linked immunosorbent assay (ELISA) using commercial kits (DRG Cortisol ELISA EIA-1887 and DRG Estradiol ELISA EIA-2693; *DRG Instruments GmbH*^®^, Marburg, Germany) following the manufacturer’s protocols.

### 2.6. Statistical Analysis

Statistical analyses were performed using JMP^®^ (Version 19.0, SAS Institute Inc., Cary, NC, USA). Continuous data were evaluated for normality and homoscedasticity using the Shapiro–Wilk and Levene’s tests, respectively. Non-normally distributed continuous variables (farrowing duration and expulsion intervals) were log_10_-transformed prior to analysis to meet model assumptions of normality and homoscedasticity of residuals, and were analyzed using a Linear Mixed Model (LMM). These data are presented in tables and text as unadjusted arithmetic means ± standard error (SE).

Discrete sow performance traits (total born, born alive, stillborn, and mummified piglets) were analyzed using a Generalized Linear Mixed Model (GLMM) assuming a Poisson distribution with a log-link function for count data.

Both LMM and GLMM included the fixed effects of treatment (G1 to G5), parity (primiparous vs. multiparous), and their interaction (treatment × parity). Sow nested within treatment was included as a random effect in all models.

Hormonal concentrations (cortisol and estradiol) and behavioral frequencies were analyzed using a mixed model for repeated measures. The model included the fixed effects of treatment, parity, time (sampling point), and all two-way and three-way interactions. The individual sow was included as a random effect with a residual covariance structure to account for repeated evaluations over time.

For all models, post hoc pairwise comparisons of the means were performed using Tukey’s Honestly Significant Difference (HSD) test. Statistical significance was set at *p* < 0.05.

## 3. Results

Table 1 summarizes the reproductive performance variables of the sows during parturition across the evaluated treatments. Additionally, Table 2 presents the effects of parity and treatment (full-dose versus split-dose administration of PGF2α, with or without caffeine) on litter characteristics at birth. Individual raw data for farrowing duration across all 50 animals is in Supplementary Table S1.

### 3.1. Farrowing Duration

Farrowing duration was significantly affected by the treatment (*p* = 0.002), whereas no significant main effect of parity (*p* = 0.589) or treatment × parity interaction (*p* = 0.812) was observed (Table 1). Sows in the PGF2α SD and PGF2α SD + Caffeine groups exhibited the shortest farrowing durations, significantly reducing farrowing time compared to the Control, PGF2α FD, and PGF2α FD + Caffeine groups. No significant differences in farrowing duration were observed among the Control, PGF2α FD, and PGF2α FD + Caffeine treatments (Table 1).

### 3.2. Birth Intervals

Regarding multiparous sows, only the PGF2α SD and PGF2α SD + Caffeine groups exhibited significant differences compared to the PGF2α FD group due to the treatment effect (*p* = 0.0042), while remaining statistically similar to the other study groups. In primiparous sows, the PGF2α SD and PGF2α SD + Caffeine groups showed a significantly shorter birth interval than the PGF2α FD group (*p* = 0.0201) but were similar to the remaining treatments. Furthermore, a significant interaction effect with parity was detected within the PGF2α FD treatment (*p* = 0.0491), where primiparous sows exhibited a birth interval that was up to 3.5 min longer than that of multiparous sows (Table 1).

### 3.3. Total Piglets Born (TPB)

Primiparous sows had litters ranging from 12.80 to 14.00 total piglets born (TPB), whereas multiparous sows presented between 14.75 and 15.83 TPB. Significant differences due to the main effect of parity were observed across almost all groups (*p* < 0.0001), with multiparous sows exhibiting a higher TPB, with the sole exception of the PGF2α FD group (*p* = 0.2541). Because the experimental treatments were administered only 24 h prior to the expected farrowing date, these observed differences in TPB reflect pre-existing litter characteristics rather than any treatment-induced effects (Table 2).

**Table 1:** Influence of full-dose versus split-dose administration of PGF2α, with or without caffeine, on performance indicators of induced sows.

	Parity	Control (*n* = 5 *p*, 5 M)	PGF2α FD (*n* = 5 *p*, 5 M)	PGF2α SD (*n* = 5 *p*, 5 M)	PGF2α FD + Caffeine (*n* = 5 *p*, 5 M)	PGF2α SD + Caffeine (*n* = 5 *p*, 5 M)	*p*-Tx	*p*-Parity	PTx × Parity
		Mean ± SE	Mean ± SE	Mean ± SE	Mean ± SE	Mean ± SE			
Farrowing duration (min)	*p*	200.40 ± 43.37 ^AB^ ^1^	239.40 ± 99.43 ^A^ ^1^	122.40 ± 82.24 ^B^ ^1^	237.60 ± 59.97 ^A^ ^1^	136.00 ± 38.33 ^B^ ^1^	0.002	0.589	0.812
	M	215.40 ± 87.11 ^AB^ ^1^	234.00 ± 35.08 ^A^ ^1^	134.80 ± 73.84 ^C^ ^1^	220.40 ± 50.81 ^A^ ^1^	158.00 ± 44.82 ^B^ ^1^			
Expulsion interval (min)	*p*	14.02 ± 2.13 ^AB^ ^1^	19.68 ± 2.89 ^A^ ^1^	12.89 ± 1.97 ^B^ ^1^	15.57 ± 2.26 ^AB^ ^1^	9.65 ± 2.13 ^B^ ^1^	0.0042	0.0201	0.0491
	M	13.34 ± 1.69 ^AB^ ^1^	16.11 ± 1.95 ^B^ ^2^	10.40 ± 1.03 ^A^ ^1^	14.34 ± 1.64 ^BA^ ^1^	12.52 ± 1.05 ^A^ ^1^			

Data are presented as unadjusted arithmetic means ± standard error (SE). *n* = 5 sows per treatment group for primiparous (*p*) and *n* = 5 for multiparous (M) sows. PGF2α FD: Full dose PGF2α; PGF2α SD: Split dose PGF2α. ^A,B,C^ Means within a row with different superscript letters differ significantly (*p* < 0.05). ^1,2^ Different superscript numbers denote significant differences among parities for the same treatment (*p* < 0.05).

**Table 2:** Litter characteristics at birth in sows subjected to full-dose versus split-dose administration of PGF2α, with or without caffeine.

	Parity	Control (*n* = 5 *p*, 5 M)	PGF2α FD (*n* = 5 *p*, 5 M)	PGF2α SD (*n* = 5 *p*, 5 M)	PGF2α FD + Caffeine (*n* = 5 *p*, 5 M)	PGF2α SD + Caffeine (*n* = 5 *p*, 5 M)	*p*-Tx	*p*-Parity	PTx × Parity
		Mean ± SE	Mean ± SE	Mean ± SE	Mean ± SE	Mean ± SE			
Total number of piglets born per litter (*n*)	*p*	12.8 ± 0.7 A ^2^	14.0 ± 0.5 A ^1^	13.6 ± 0.4 A ^2^	13.4 ± 0.3 A ^2^	13.6 ± 0.2 A ^2^	0.8120	<0.0001	0.2541
	M	15.25 ± 1.86 A ^1^	14.75 ± 1.86 A ^1^	15.83 ± 0.52 A ^1^	15.6 ± 1.67 A ^1^	15 ± 1.12 A ^1^			
Number of piglets born alive per litter (*n*)	*p*	11.8 ± 0.5 A ^1^	12.8 ± 0.6 A ^1^	13.0 ± 0.4 A ^1^	13.0 ± 0.71 A ^1^	13.00 ± 0.9 A ^1^	0.1245	0.0814	0.0036
	M	13.75 ± 1.64 C ^1^	12.5 ± 1.64 B ^1^	13.66 ± 1.34 C ^1^	13.6 ± 1.41 C ^1^	14.66 ± 1.34 A ^1^			
Number of stillborn (*n*)	*p*	0.89 ± 0.1 A ^1^	0.99 ± 0.19 A ^2^	0.69 ± 0.15 AB ^1^	0.42 ± 0.15 B ^2^	0.49 ± 0.1 B ^1^	<0.0001	<0.0001	<0.0001
	M	1.3 ± 0.11 B ^1^	1.6 ± 0.09 C ^1^	0.8 ± 0.3 A ^1^	1 ± 0.45 BC ^1^	0.4 ± 0.12 A ^1^			
Number of mummified fetuses (*n*)	*p*	0.21 ± 0.01 C ^1^	0.25 ± 0.1 D ^1^	0.12 ± 0.4 B ^1^	0 ± 0.0 A ^2^	0.24 ± 0.1 D ^1^	0.0115	0.1420	0.0001
	M	0.1 ± 0.16 A ^1^	0.25 ± 0.26 A ^1^	0.66 ± 0.21 A ^1^	0 ± 0.23 A ^1^	0.33 ± 0.21 A ^1^			

Data are presented as unadjusted arithmetic means ± standard error (SE). *n* = 5 sows per treatment group for primiparous (*p*) and *n* = 5 for multiparous (M) sows. PGF2α FD: Full dose PGF2α; PGF2α SD: Split dose PGF2α. Total bor*n* = total piglets born per litter; born alive = piglets born alive per litter; stillbor*n* = stillborn piglets per litter; mummified = mummified fetuses per litter. ^A,B,C,D^ Means within a row with different superscript letters differ significantly (*p* < 0.05). ^1,2^ Different superscript numbers denote significant differences among parities for the same treatment (*p* < 0.05).

### 3.4. Piglets Born Alive (PBA)

Regarding the number of piglets born alive, primiparous sows showed no significant differences due to the treatment effect (*p* = 0.1245). Conversely, multiparous sows exhibited significant variations driven by the treatments (*p* = 0.0036), with the PGF2α SD + Caffeine group achieving the highest number of piglets born alive. No significant differences were observed due to the main effect of parity (*p* = 0.0814) (Table 2).

### 3.5. Stillborns

For multiparous sows, a significant treatment effect was observed, the PGF2α SD and PGF2α SD + Caffeine groups exhibited a lower number of stillborn piglets, in contrast to the PGF2α FD group, which presented the highest incidence (*p* < 0.0001). When analyzing the impact of parity in primiparous sows, the lowest incidence of stillborn piglets was observed in the treatments involving PGF2α FD + Caffeine and PGF2α SD + Caffeine, which performed similarly to the PGF2α SD group, whereas the PGF2α FD group showed the highest incidence (*p* < 0.0001). Additionally, a significant interaction effect between parity and treatment was detected for both the PGF2α FD (*p* < 0.0001) and PGF2α FD + Caffeine (*p* < 0.0001) groups, where multiparous sows consistently exhibited a higher number of stillborn piglets than primiparous sows (Table 2).

### 3.6. Mummified Fetuses

A significantly higher occurrence of mummified piglets was detected in primiparous sows assigned to the PGF2α FD and PGF2α SD + Caffeine treatments compared to all other evaluated groups (*p* < 0.0115). Conversely, no significant differences were detected among the multiparous sow groups (*p* = 0.1420). Only the PGF2α FD + Caffeine treatment exhibited a significant parity effect (*p* = 0.0001), with multiparous sows presenting a higher number of mummified fetuses. Notably, these variations are independent of the experimental treatments, as caffeine was administered only 24 h pre-farrowing, and the observed values remain fully aligned with the farm’s historical baseline for mummified fetuses (1.5% per litter) (Table 2).

### 3.7. Sow Behavior During Farrowing

**Figure 3:**
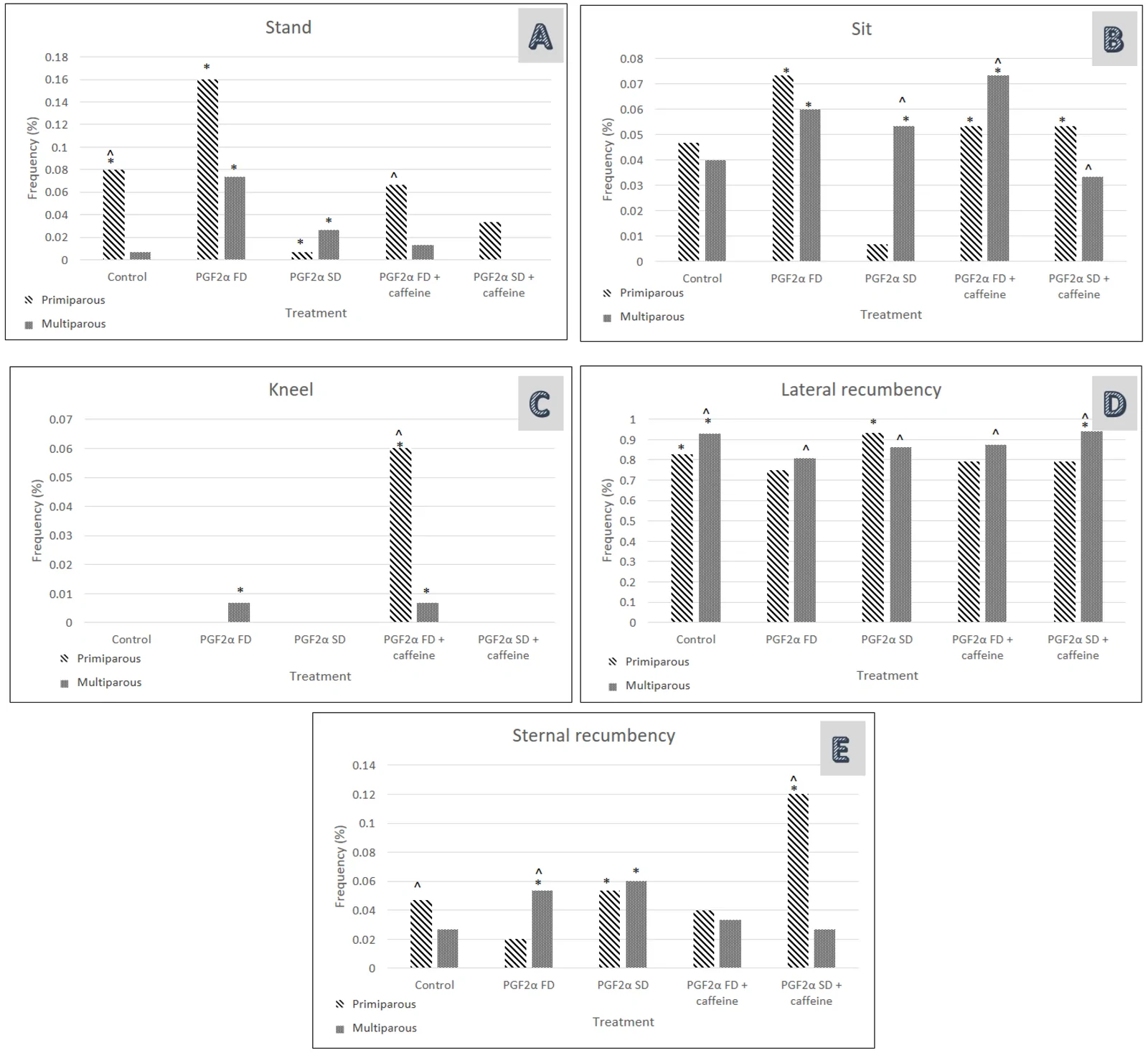
Postural change frequency in primiparous and multiparous sows during the farrowing expulsion phase, illustrated by posture: (**A**) Stand, (**B**) Sit, (**C**) Kneel, (**D**) Lateral recumbency, and (**E**) Sternal recumbency. Frequencies of each evaluated postures are presented across the experimental groups. Asterisks (*) indicate significant differences among treatment groups within the same parity (Tukey’s HSD, *p* < 0.05). Carets (^) indicate significant differences between parities within the same treatment (*p* < 0.05). PGF2α, prostaglandin F2α; FD, Full Dose; SD, Split Dose. Total sample size *n* = 50 (*n* = 10 per treatment; *n* = 25 per parity).

Figure 3 displays the behavioral frequencies evaluated across the study groups during the farrowing expulsion phase.

As shown in Figure 3A, the standing posture frequency was significantly higher in primiparous sows from the Control and PGF2α FD groups compared to the other treatments (*p* < 0.05). Among multiparous sows, the PGF2α FD and PGF2α SD groups adopted this posture most frequently (*p* < 0.05). Regarding the main effect of parity, the Control, PGF2α FD, and PGF2α FD + Caffeine groups exhibited a higher frequency of standing behavior (*p* < 0.05).

Figure 3B details the frequencies corresponding to the sitting posture. In primiparous sows, the PGF2α FD, PGF2α FD + Caffeine, and PGF2α SD + Caffeine groups showed the highest frequencies (*p* < 0.05). For multiparous sows, the treatments that elicited this posture most frequently were PGF2α FD, PGF2α SD, and PGF2α FD + Caffeine (*p* < 0.05). Significant variations due to parity were observed in the PGF2α SD, PGF2α FD + Caffeine, and PGF2α SD + Caffeine groups (*p* < 0.05).

Figure 3C presents the frequency of the kneeling posture, which was the least common behavior among all those evaluated. In primiparous sows, only the PGF2α FD + Caffeine group displayed this posture (*p* < 0.05). For multiparous sows, the PGF2α FD and PGF2α FD + Caffeine groups performed this posture (*p* < 0.05). Regarding the parity effect, primiparous sows from the PGF2α FD + Caffeine group exhibited this behavior more frequently than their multiparous counterparts (*p* < 0.05).

Figure 3D illustrates the frequency with which the sows maintained the lateral recumbency (lying lateral) posture. In primiparous sows, the Control and PGF2α SD groups adopted this posture more frequently compared to the other treatments (*p* < 0.05). In multiparous sows, those in the Control and PGF2α SD + Caffeine groups presented this posture with greater frequency (*p* < 0.05). Regarding the effect of parity, all evaluated groups showed significant differences (*p* < 0.05), with multiparous sows consistently maintaining this posture more frequently across all treatments.

Finally, Figure 3E presents the data for the sternal recumbency (lying ventral) posture. In primiparous sows, the PGF2α SD + Caffeine group, followed by the PGF2α SD group, showed the highest frequency (*p* < 0.05). In multiparous sows, the PGF2α FD and PGF2α SD groups maintained this posture more frequently than the other study groups (*p* < 0.05). Regarding parity-driven differences, significant effects were found in the Control, PGF2α SD + Caffeine, and PGF2α FD groups; primiparous sows adopted this posture more frequently in the first two groups, whereas multiparous sows did so in the latter (*p* < 0.05).

### 3.8. Serum Cortisol Concentrations

**Figure 4:**
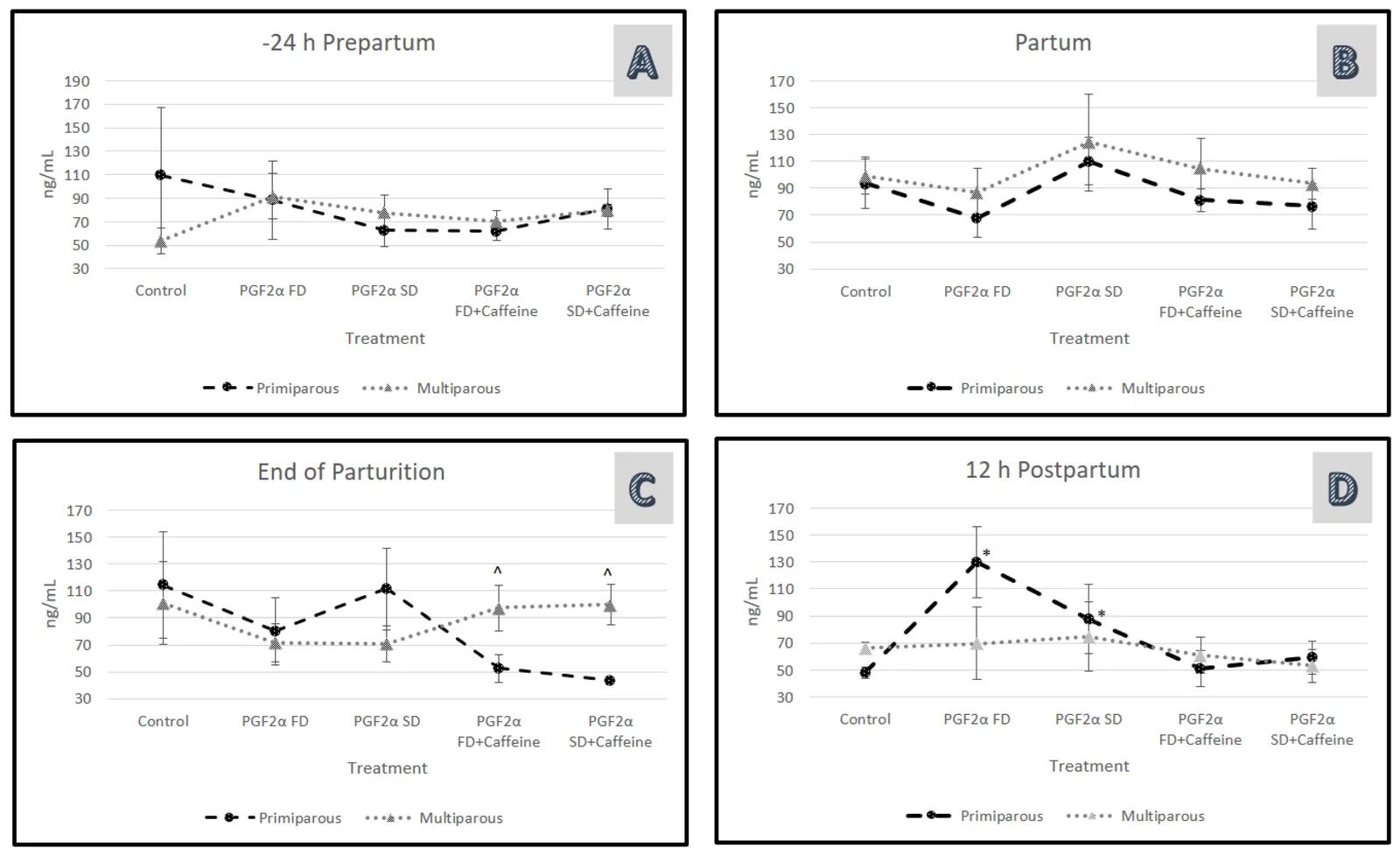
Serum cortisol concentrations (ng/mL) pre-, during, and postpartum in primiparous and multiparous sows induced to farrow with PGF2α and administered caffeine. Data are presented across all study groups and sampling points. Asterisks (*) denote significant differences among treatment groups within the same parity (Tukey’s HSD, *p* < 0.05). Carets (^) indicate significant differences between parities within the same treatment (*p* < 0.05). PGF2α, prostaglandin F2α; FD, Full Dose; SD, Split Dose. Total sample size *n* = 50 (*n* = 10 per treatment; *n* = 25 per parity).

Figure 4 presents the serum cortisol concentrations of sows induced to farrow, with and without caffeine administration, evaluated at four different time points. At 24 h prepartum, no significant differences were observed (*p* > 0.05) among treatments in either primiparous or multiparous sows; these uniform values served strictly as a physiological baseline prior to treatment administration. No differences related to the main effect of parity were observed at this time point (*p* > 0.05). Similarly, at the onset of farrowing, no significant differences associated with the treatments were detected. At the completion of farrowing, treatment effects remained non-significant; however, a significant parity effect was observed (*p* < 0.05), with the lowest cortisol levels observed specifically in primiparous sows from the PGF2α FD + Caffeine and PGF2α SD + Caffeine groups. At 12 h postpartum, primiparous sows from the PGF2α FD and PGF2α SD groups showed the highest cortisol levels (*p* = 0.0192). Conversely, no significant treatment or parity effects were found in multiparous sows at this final sampling point.

Figure 4 shows the serum cortisol concentrations (ng/mL) pre-, during, and postpartum in primiparous and multiparous sows induced to farrow with PGF2α and administered caffeine.

### 3.9. Serum Estradiol Concentrations

**Figure 5:**
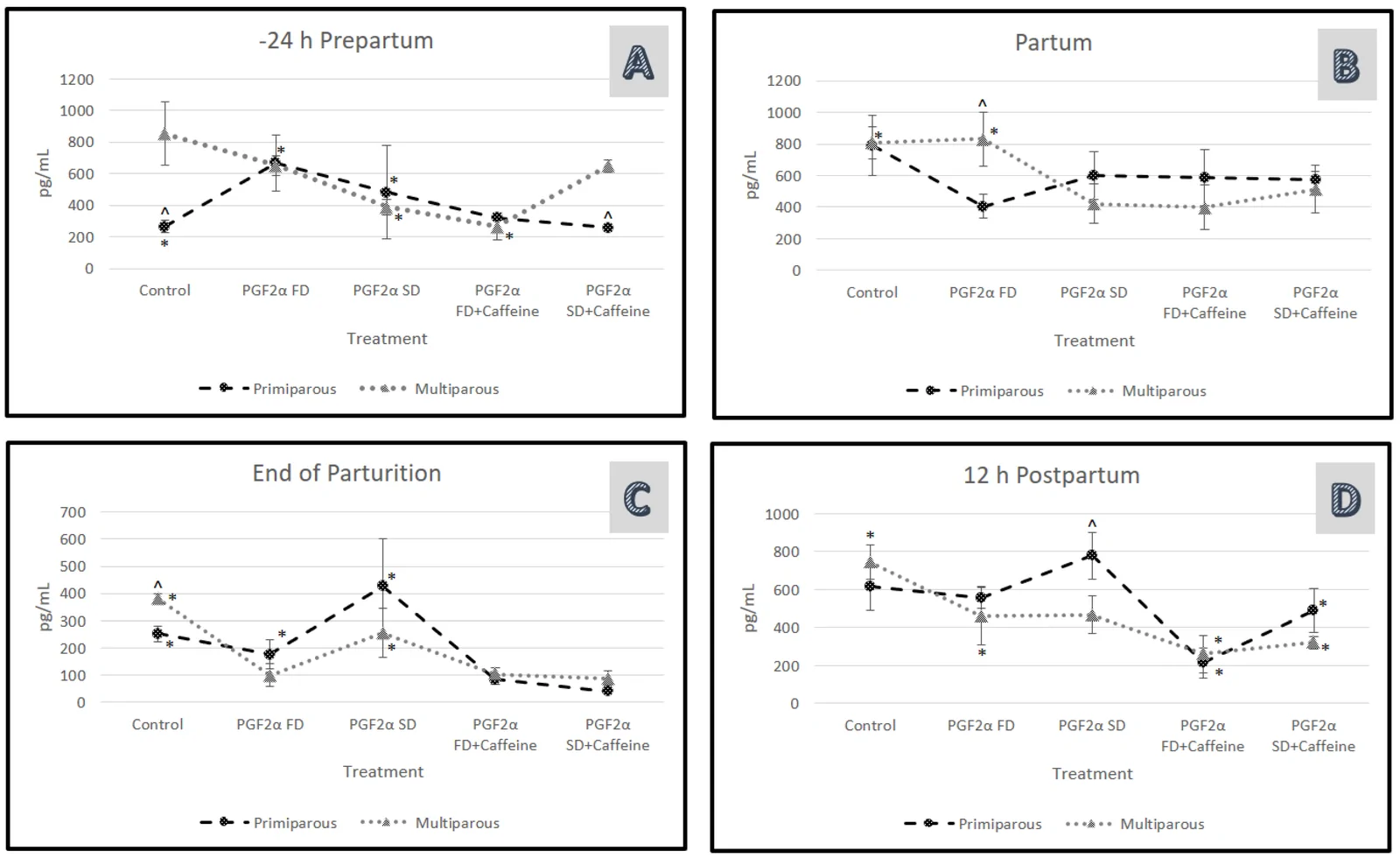
Serum estradiol concentrations (pg/mL) pre-, during, and postpartum in primiparous and multiparous sows induced to farrow with PGF2α and administered caffeine. Data are presented across all study groups and sampling points. Asterisks (*) denote significant differences among treatment groups within the same parity (Tukey’s HSD, *p* < 0.05). Carets (^) indicate significant differences between parities within the same treatment (*p* < 0.05). PGF2α, prostaglandin F2α; FD, Full Dose; SD, Split Dose. Total sample size *n* = 50 (*n* = 10 per treatment; *n* = 25 per parity).

Figure 5 presents the serum estradiol concentrations of sows induced to farrow, with and without caffeine administration, evaluated at four specific time points. At 24 h prepartum within primiparous sows, the PGF2α FD group presented the highest estradiol concentrations compared to the other treatments (*p* < 0.05), except for the PGF2α SD group, which showed no significant differences. In multiparous sows, the Control, PGF2α FD, and PGF2α SD + Caffeine groups exhibited the highest concentrations (*p* < 0.05). These baseline differences in estradiol levels at 24 h prepartum were unrelated to the treatments, as interventions had not yet occurred; thus, these variations likely reflect individual physiological differences among the sows. Regarding parity effects at this time point, significant differences were found only in the Control and PGF2α SD + Caffeine groups, with multiparous sows displaying higher concentrations than primiparous sows.

At the onset of farrowing, no treatment-driven differences were found in primiparous sows (*p* > 0.05). Conversely, in multiparous sows, the Control and PGF2α FD groups achieved the highest estradiol concentrations (*p* < 0.05), while the remaining treatments performed similarly. Furthermore, a significant parity effect was detected only within the PGF2α FD group (*p* < 0.05), where multiparous sows exhibited higher estradiol concentrations than primiparous sows.

At the completion of farrowing, the highest concentrations in primiparous sows were observed in the Control, PGF2α FD, and PGF2α SD groups compared to the other treatments (*p* < 0.05). Similarly, in multiparous sows, the highest concentrations were detected in the Control and PGF2α SD groups (*p* < 0.05). However, a significant parity effect at farrowing completion was observed exclusively in the Control group, with multiparous sows showing higher levels than primiparous sows (*p* < 0.05).

At 12 h postpartum, primiparous sows from the PGF2α FD + Caffeine group presented the lowest estradiol concentrations compared to the other treatments, although they were statistically similar to those of the PGF2α SD + Caffeine group (*p* < 0.05). In multiparous sows, the Control group exhibited the highest concentrations, which were significantly greater than all other groups, with the sole exception of the PGF2α FD group (*p* < 0.05). Finally, a significant parity effect at this post-farrowing point was detected only in the PGF2α SD group, with higher values observed in primiparous sows (*p* < 0.05).

## 4. Discussion

Modern genetic improvement in swine production has significantly increased litter size [15], with averages of 16 piglets being common [16,17], and prolific lines reaching up to 23 piglets per litter [18]. This marked increase in prolificacy has altered farrowing kinetics, prolonged total parturition duration, and compromised maternal performance during farrowing [19,20,21,22].

This scenario, compounded by the physiological and anatomical disparities between primiparous and multiparous sows [23,24], contributes to a high variability in reproductive performance within commercial herds [7].

Our findings indicate that the time required for farrowing was significantly altered by the specific induction method (*p* = 0.002), regardless of whether the sows were primiparous or multiparous, whereas caffeine administration showed no direct modifying effect. This observation aligns with previous findings [4] reporting that farrowing duration is unaffected by caffeine. Specifically, the PGF2α SD and PGF2α SD + Caffeine treatments shortened total farrowing duration across both parity groups, achieving reductions of up to 100 min compared to the full-dose induction groups (PGF2α FD and PGF2α FD + Caffeine) and control sows.

These results corroborate the findings of Tospitakkul et al. [25], who reported a reduction of up to 50 min in farrowing duration using the split-dose (SD) technique compared to single full-dose (FD) induction (*p* < 0.05). This outcome is likely linked to the fact that dual administration of PGF2α significantly minimizes the variability in gestation length. Given that the endogenous release of PGF2α is inherently pulsatile [26], a single injection may fail to trigger complete and synchronous luteolysis in all corpora lutea. In complete agreement with this mechanism, certain sows fail to fully respond to a single prostaglandin injection (FD) due to incomplete luteolysis, which allows specific corpora lutea to recover, thereby delaying or prolonging the active farrowing process [8]. Conversely, the split-dose (SD) technique—applying two lower doses at a 6 h interval—effectively mimics natural pulsatile PGF2α concentrations, maximizing the luteolytic effect and ensuring a more efficient and coordinated onset of parturition [27]. When this robust luteolysis is combined with caffeine, the protocol achieves a distinct synergistic advantage over the FD + Caffeine combination, providing tighter synchronization and more predictable farrowing kinetics. Additionally, cloprostenol exhibits a high affinity for PGF2α receptors and a prolonged half-life of approximately 3 h, sustaining its uterotonic and luteolytic stimuli over an extended period; however, whether cloprostenol is inherently more effective at corpus luteum lysis remains a subject of debate [28].

Unlike the findings of Björkman et al. [29] and Ju et al. [30], who reported that farrowing duration increases with parity—noting that second-parity sows averaged 273 ± 147 min, whereas fifth-parity sows reached 481 ± 278 min—the sows in our study did not show significant variations in farrowing duration due to parity (*p* = 0.589). This lack of difference aligns with the results of Schulthess et al. [31], who observed no parity-related differences between low-parity (parities 1–4; 231.1 ± 101.1 min) and high-parity sows (≥5; 227.4 ± 63.5 min). Similarly, across all experimental groups, the shortest birth intervals were consistently recorded in the PGF2α SD and PGF2α SD + Caffeine groups. This strongly implies that the induction method dictated these kinetics, as the split-dose protocol extends the window of cloprostenol stimulus owing to its 3 h half-life [8].

Regarding maternal behavior, posture changes in primiparous sows were widely distributed among all evaluated behaviors. In contrast, lateral recumbency and sitting predominated in multiparous sows. These behavioral profiles are likely influenced not only by previous maternal experience [23,32], but also by circulating cortisol concentrations [33] and the overall ease of farrowing [34]. This correlation is particularly relevant since primiparous sows from the Control and PGF2α FD groups exhibited the highest cortisol levels and the highest frequency of standing posture across all treatments and parities (*p* < 0.05). Furthermore, these full-dose induction groups experienced the longest farrowing durations. Optimal maternal behavior in crated sows is characterized by relative passivity, fewer posture changes [20], and the maintenance of lateral recumbency [23,32]; however, notable disparities exist in stress susceptibility and maternal responses at parturition between primiparous and multiparous females [35,36].

Lateral recumbency is considered the physiological norm during the expulsion phase in swine [37]. In this study, multiparous sows maintained this posture more frequently, likely due to better habituation to the farrowing crate environment and past experience. Conversely, primiparous sows showed a significantly lower frequency of lateral recumbency, indicating a higher vulnerability to environmental stressors [36]. These primiparous females also exhibited the highest frequency of the standing posture, driven by the heightened distress and restlessness associated with their first farrowing experience [38]. Primiparous sows are known to exhibit elevated cortisol levels at the onset of farrowing [13], which directly correlates with more frequent posture changes, a pattern that coincides with observations in pre-farrowing sows [23,39].

Notably, primiparous sows in the PGF2α SD group performed fewer posture changes (standing, sitting, and kneeling) and maintained a higher frequency of sternal and lateral recumbency. This behavior could be a direct benefit of the split-dose induction, which shortens total farrowing time [8]. Likewise, a higher frequency of lateral recumbency is associated with a greater ease of farrowing and lower physical exhaustion [34].

Mean cortisol levels in multiparous sows tended to be slightly higher during the prepartum period, onset, and completion of farrowing. This finding agrees with Hales et al. [40] and Ison et al. [13], who attributed these higher concentrations to prolonged farrowing durations often associated with larger litter sizes in older females. However, at 12 h postpartum, the highest cortisol concentrations were observed in primiparous sows, suggesting a superior adaptive and recovery capacity in multiparous sows due to their physiological maturity and previous experience [17].

Fluctuations in systemic cortisol levels are multifactorial, driven by farrowing duration, parity, environment, and the specific induction protocol. In our study, the full-dose protocol resulted in a farrowing duration up to 100 min longer than the split-dose treatments, mirroring the 50 min difference reported by Tospitakkul et al. [25], parity remains a critical determinant, as primiparous sows manifest greater distress when facing parturition as a novel event [7]. Furthermore, individual physiological variations within the same group cannot be ruled out [41]. Natural circadian rhythms regulating cortisol secretion could have also influenced these peripartum endocrine dynamics [42]. Finally, farrowing induction itself is known to alter the normal surge of peripartum cortisol [43] by acutely modifying farrowing kinetics and uterine pressure [44].

Regarding serum estradiol, substantial variability was observed even prior to treatment application. This baseline variation may relate to individual litter size characteristics [45] or intrinsic physiological differences among individual sows, representing normal variations within the physiological range. It is also possible that some inductions occurred slightly more than 24 h prior to spontaneous farrowing due to minor recording errors at insemination or natural variations in individual gestation length [25]. Estradiol concentrations are tightly linked to the exact temporal progress of gestation [46,47,48]; therefore, exogenous hormonal induction can alter natural endocrine profiles [44,48], particularly under the split-dose protocol due to repeated applications. Because primiparous sows are still growing and have not achieved full reproductive and musculoskeletal maturity [49], their endocrine profiles logically differ from those of multiparous sows. Nevertheless, the post-farrowing decline in estrogen levels observed across all groups represents an expected physiological clearance, with values remaining within the normal ranges described in the literature [38,50].

The results of the present study highlight a few limitations that could be addressed in future research. In summary, the main limitations of this study include the lack of long-term evaluation on piglet development and survival, the omission of an uninduced caffeine-only control group due to logistical and protocol priorities, and the 12 h limit on post-farrowing endocrine monitoring.

## 5. Conclusions

The results of the present study suggest that farrowing induction using a split-dose protocol (PGF2α SD) combined with caffeine administration is an effective strategy to optimize parturition performance in swine. The PGF2α SD + Caffeine treatment significantly reduced farrowing duration and birth intervals across both parities, thereby increasing the number of piglets born alive and minimizing perinatal mortality, particularly in multiparous sows. Furthermore, this protocol decreased the frequency of posture changes commonly associated with discomfort in crated sows (such as standing or sitting), while promoting a higher proportion of lateral recumbency in multiparous sows and sternal recumbency in primiparous sows. Finally, peripartum serum cortisol and estradiol concentrations demonstrated that the maternal endocrine response during farrowing is heavily dictated by the female’s parity. Consequently, the combination of PGF2α SD + Caffeine represents a key management strategy to improve farrowing efficiency, synchronization, and maternal welfare in commercial swine production units.

## Data Availability

The original contributions presented in this study are included in the article/Supplementary Material. Further inquiries can be directed to the corresponding authors.

## Supplementary Materials

The following supporting information can be downloaded at: https://www.mdpi.com/article/10.3390/vetsci13080772/s1, Table S1:Individual raw data for farrowing duration across all 50 animals.

## Abbreviations

The following abbreviations are used in this manuscript:

PGF2α	Prostaglandin F2 alpha
FD	Full Dosis
SD	Split Dosis
